# Molecular Mechanisms of Endothelialitis in SARS-CoV-2 Infection: Evidence for VE-Cadherin Cleavage by ACE2

**DOI:** 10.3390/ijms241512525

**Published:** 2023-08-07

**Authors:** Laurence Bouillet, Alban Deroux, Meryem Benmarce, Chloé Guérin, Laura Bouvet, Olivia Garnier, Donald K. Martin, Isabelle Vilgrain

**Affiliations:** 1 University Grenoble Alpes, CNRS, TIMC-IMAG/T-RAIG (UMR 5525), 38000 Grenoble, France; lbouillet@chu-grenoble.fr (L.B.);; 2 Grenoble Hospital Grenoble Alpes (CHUGA), University Grenoble Alpes, 38000 Grenoble, France; aderoux@chu-grenoble.fr; 3 Internal Medicine, University Hospital Centre Grenoble Alpes, CEDEX 9, 38043 Grenoble, France; cguerin@chu-grenoble.fr; 4 University Grenoble Alpes, CNRS, TIMC-IMAG/SyNaBi (UMR 5525), 38000 Grenoble, Francedonald.martin@univ-grenoble-alpes.fr (D.K.M.); 5 University Grenoble Alpes, INSERM U13, CEA, Institute of Interdisciplinary Research of Grenoble (IRIG), Laboratory of Biosciences et Bioingénierie Pour la Santé (BGE)-Biomics, 38000 Grenoble, France

**Keywords:** endothelium, VE-cadherin, ACE2, SARS-CoV-2 infection, long COVID syndrome

## Abstract

Long COVID-19 syndrome appears after Severe Acute Respiratory Syndrome-Corona Virus (SARS-CoV-2) infection with acute damage to microcapillaries, microthrombi, and endothelialitis. However, the mechanisms involved in these processes remain to be elucidated. All blood vessels are lined with a monolayer of endothelial cells called vascular endothelium, which provides a the major function is to prevent coagulation. A component of endothelial cell junctions is VE-cadherin, which is responsible for maintaining the integrity of the vessels through homophilic interactions of its Ca^++^-dependent adhesive extracellular domain. Here we provide the first evidence that VE-cadherin is a target in vitro for ACE2 cleavage because its extracellular domain (hrVE-ED) contains two amino acid sequences for ACE2 substrate recognition at the positions ^256^P-F^257^ and ^321^PMKP-^325^L. Indeed, incubation of hrVE-ED with the active ectopeptidase hrACE2 for 16 hrs in the presence of 10 μM ZnCl_2_ showed a dose-dependent (from 0.2 ng/μL to 2 ng/μL) decrease of the VE-cadherin immunoreactive band. In vivo, in the blood from patients having severe COVID-19 we detected a circulating form of ACE2 with an apparent molecular mass of 70 kDa, which was barely detectable in patients with mild COVID-19. Of importance, in the patients with severe COVID-19 disease, the presence of three soluble fragments of VE-cadherin (70, 62, 54 kDa) were detected using the antiEC1 antibody while only the 54 kDa fragment was present in patients with mild disease. Altogether, these data clearly support a role for ACE2 to cleave VE-cadherin, which leads to potential biomarkers of SARS-CoV-2 infection related with the vascular disease in “Long COVID-19”.

## 1. Introduction

The world continues to deal with successive waves of the Coronavirus disease 2019 (COVID-19), for which often disabling sequelae are called “post-COVID-19 syndrome” [1]. It has a worldwide impact and was first diagnosed in 2019, in Wuhan, China [2]. Up to now, more than 280 million cases have been confirmed, leading to over 5.4 million deaths in the world [3]. Among the distinctive features of COVID-19 are the associated vascular changes, which are due to direct endothelial infection. Several studies have reported the onset of vasculitis syndromes in COVID-19 patients, with histologic evidence also in the liver, lung, skin, or kidney. The first evidence was reported by Varga et al. [4] who found viral bodies in endothelial cells (ECs) from glomerular capillary loops of the transplanted kidney of a patient, and endothelialitis in an additional two patients. Meanwhile, Ackermann et al. [5] have observed morphologic and molecular changes in the peripheral lung of patients who died from COVID-19, which consisted of severe endothelial injury, widespread thrombosis with microangiopathy, and vascular angiogenesis. Chhetri et al. [6] showed that many COVID-19 patients exhibited severe metabolic acidosis, which indicated possible microcirculation dysfunction. Therefore, understanding the mechanism of endothelial dysfunction in COVID-19 would help to understand the basis for vascular damage during long COVID syndrome and so lead to better clinical care for patients.

The integrity of the dense vascular network surrounding small pulmonary alveoli depends upon endothelial adherens junctions (EJ) and the pericyte coverage of the small capillaries. As far as endothelial cells are concerned, the role of the transmembrane protein called VE-cadherin is crucial in maintaining the tight association between ECs. The whole VE-cadherin structure maintains adhesion of ECs by homotypic interactions involving the hydrophobic two amino acids (W49 and W51) of the EC1 domain (110 residues) [7,8]. The cytoplasmic domain of the protein is bound with the catenins that form the structural bridges with the actin cytoskeleton to enhance adhesion. Because of these VE-cadherin interactions, the EJ are extremely stable in adults except upon challenge by inflammatory mediators, such as histamine, thrombin, VEGF, TNF-α, and platelet-activating factor [9,10,11,12,13]. In previous studies we and others have demonstrated that disruption of cell-cell junctions depends on intracellular kinases and/or phosphatases [8]. VE-cadherin tyrosine phosphorylation is a mechanism involved in weakening the endothelial cell barrier resulting from cell stimulation by growth factors and inflammatory cytokines. Indeed, the post-translational modification creates structural changes in the protein conformation, which is more sensitive to proteases and thus the phosphorylation precedes the cleavage of its extracellular domain (sVE) that is found in the human blood [13].

A clinical example is Hereditary Angioedema (HAE), which is characterized by recurrent episodes of edema that can be life-threatening if not treated properly [14,15]. HAE is an autosomal-dominant inherited disease caused by C1 inhibitor deficiency as a result of mutations in the SERPING1 gene. Because of C1 inhibitor deficiency in HAE, the plasma proteolytic cascades are activated during an attack (plasma Kallikrein and coagulation factor XIIa) and kinins are generated. Among these, Bradykinin has been shown to be the predominant mediator of enhanced vascular permeability in HAE attacks where the patients present recurrent swellings. Exploring whether VE-cadherin was involved in the pathogenesis of HAE, we demonstrated that Bradykinin and Kallikrein induced a release of the 90 and 75-Da fragments [14]. The direct proteolytic action of Kallikrein occurred at two consensus sites of cleavage at the peptide bonds ^411^R-T^412^ and ^565^R-T^566^ in the VE-cadherin extracellular adhesive domain (sequence identifier, P03952). The 90 kDa VE-cadherin extracellular domain was detected in the patient’s serum during the attack, whereas it was barely detectable before and after the attack. Altogether, these data collectively support that the detection of a soluble VE-cadherin fragment might have clinical interest as a potential biomarker for the diagnosis. As reported by Mehta et al. and others [16,17], a cytokine storm results from the SARS-CoV-2 infection, leading to increased capillary leakage of fluid, and recruitment of immune-inflammatory cells in lungs. Studies of Long COVID patients have shown persistent deregulation of a broad range of cytokines long after infection [1] and many vascular changes affect the entire pulmonary vascular tree, from large-calibre vessels to capillaries. Altogether, these data suggest that the EJ and VE-cadherin might have a role in the pathogenesis of Long COVID.

To support this hypothesis, the major protease Angiotensin Converting Enzyme-2 (ACE2) is a potential candidate to have a direct proteolytic action on VE-cadherin. Indeed ACE2 was shown to be expressed in different cell types including ECs [18,19,20]. Of great significance, ACE2 is an ecto-metallopeptidase orientated with its catalytic site facing the extracellular space. Its site of cleavage has been mapped and corresponds to the amino acid sequence PMKP–L, where the bond P-L is cleaved [21]. Furthermore, an active soluble ACE2 has been detected in the plasma of patients with heart failure [22,23]. To the best of our knowledge, VE-cadherin cleavage has never been studied in COVID-19 disease. Importantly, we found two consensus sites of cleavage for ACE2 at the peptide bonds (^266^PF^267^) and (^321^PMKP–L^325^) in the VE-cadherin adhesive extracellular domain. Thus, the goal of this study was to examine the effect of ACE2 on VE-cadherin extracellular domain, and to analyze ACE2 and VE-cadherin in blood from COVID-19 patients with mild or severe disease in relation to disease activity score. These data shed light on the possible enigma of the microcirculation dysfunction in COVID-19 disease.

## 2. Results

### 2.1. The EJ Are Cohesive Due to the Adhesive Properties of the Extracellular Domain of VE-Cadherin

**Figure 1:**
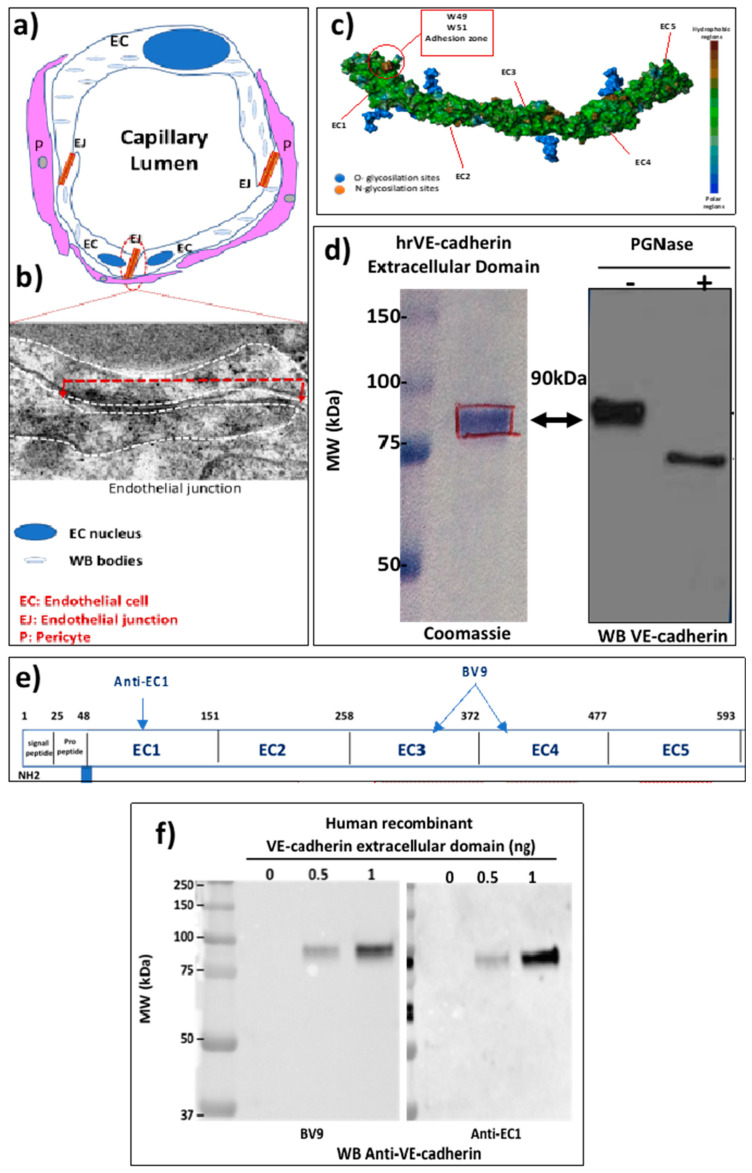
The EJ are composed of a transmembrane protein (VE-cadherin). (**a**) Schematic representation of a capillary composed of three ECs. The capillary is surrounded by pericytes. The overlap of two ECs is the EJ. (**b**) Electron microscopy of the endothelial junction. The dashed white line delineates two ECs. (**c**) Extracellular domain of VE-cadherin showing the hydrophobic amino acid W49 and W51 responsible for cohesion of ECs. (**d**) Coomassie-stained SDS-PAGE showing that human recombinant extracellular domain of VE-cadherin with an apparent mass of 90 kDa and immunoblotted with VE-cadherin antibody (clone BV9) not treated (left lane) or treated (right lane) with PGNase-F. (**e**) Schematic representation of VE-cadherin extracellular domain with the epitopes of the antibodies (Anti-EC1 and the clone BV9) against human VE-cadherin. (**f**) Immunoblotting of human recombinant VE-cadherin with the antibodies clone BV9 and anti-EC1. The positions of molecular mass standards (in kDa) are shown at the left. These experiments were repeated at least four times in a similar configuration.

Severe Acute Respiratory Syndrome-Corona Virus (SARS-CoV-2) infection causes endothelial cell injury in the COVID-19 disease leading to pulmonary pneumonia and acute respiratory distress syndrome. The pulmonary circulation is composed of pulmonary arteries and veins. Within the alveolar walls, the terminal arterioles break into a network of pulmonary capillaries. As illustrated in Figure 1a, a small capillary which can be composed of only 3 ECs maintains the blood flow through the integrity of EJ and also pericyte coverage. The ECs have pro-coagulant properties and one of the important compounds is the Von Willebrand factor, which is located in the small vesicles called Weibel-Palade bodies in the ECs (Figure 1a). The strength of ECs is due to the overlap of two ECs that can be seen in transmission electron microscopy (Figure 1b). An EJ is delineated by the dashed white line and the length of the EC junction is visualized by the red dashed line (Figure 1b). The cohesion between ECs is maintained by the adhesive transmembrane protein VE-cadherin specifically expressed in all ECs, which has a structure that allows homotypic interactions between the EC1 homologous repeats due to the presence of 2 conserved tryptophan residues in the N-terminal sequence [24] (Figure 1c). To study the protein, we next purified the human recombinant VE-cadherin ectodomain as described in Methods. It is a 90 kDa protein recognized by the monoclonal mouse anti-human VE-cadherin antibody (clone BV9) (Figure 1d, left panel). The presence of significant glycosylation sites can modify its predicted molecular mass. To further confirm this possibility the protein was subjected to a treatment with PGNase-F, which is the enzyme used for deglycosylation assays. As shown on Figure 1d, right panel, the molecular mass of the VE-cadherin was 75 kDa, which confirmed the sites of glycosylation that were mapped in the human VE-cadherin ectodomain [24]. Figure 1e illustrates the epitopes for several antibodies that can recognize this human recombinant VE-cadherin extracellular domain (hrED of VE-cadherin). These antibodies have been tested by western blot using hrED of VE-cadherin (Figure 1f). The mouse monoclonal anti-human VE-cadherin (clone BV9) recognized the recombinant protein in a dose-dependent manner (Figure 1f, left panel), which was further confirmed with the antibody directed against the domain EC1 (Figure 1f, right panel). Altogether, these tools were used to study whether VE-cadherin extracellular domain was a substrate for ACE2 in vitro.

### 2.2. VE-Cadherin Is a Direct Substrate for Angiotensin-Converting Enzyme-2 (ACE2)

**Figure 2:**
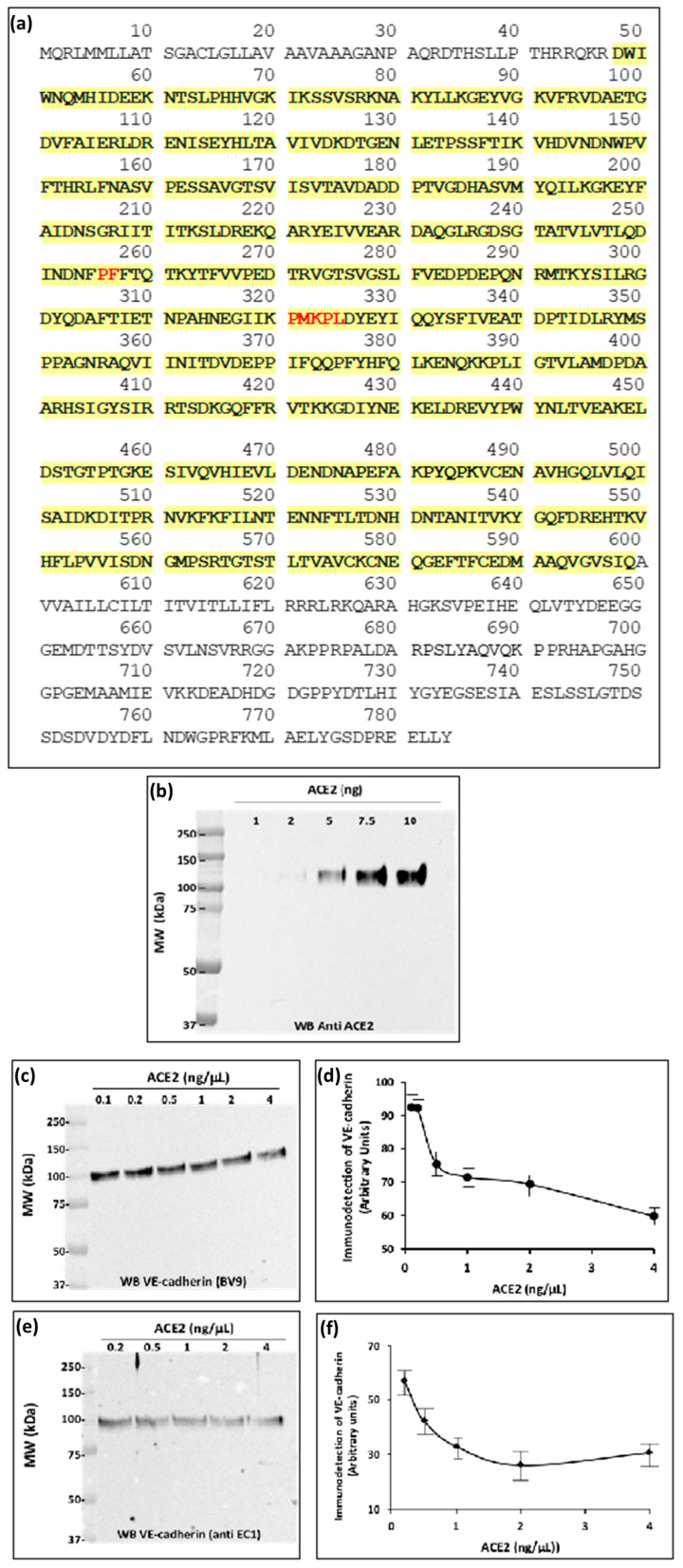
The extracellular domain of VE-cadherin is a substrate for the ectopeptidase ACE2: (**a**) VE-cadherin sequence. The yellow color highlights the amino acid sequence of the extracellular domain of the protein. The putative consensus site for the ACE2 cleavage is highlighted in red. (**b**) Representative western blot showing the human recombinant ACE2 (hrACE2) analyzed with the antibody directed against ACE2 extracellular domain. The positions of molecular mass standards (in kDa) are shown at the left (**c**,**d**). Representative western blots of VE-cadherin extracellular domain incubated overnight at room temperature with increasing concentrations of hrACE2 (0.1 to 4 ng/mL) in 50 mM 2-(N-Morpholino)-ethane sulfonic acid (MES), 300 mM NaCl, 10 μM ZnCL2, 0.01% Brig 35, pH 6.5). The samples were analyzed by SDS-PAGE and immunoblotting. The immunoreactive band was detected with the monoclonal anti VE-cadherin antibody (BV9) or the rabbit polyclonal anti-EC1 antibody (anti EC1). (**e**,**f**) Densitometric analysis of the 90 kDa band using ImageJ software showed a dose-dependent decrease in VE-cadherin band intensity. This experiment was performed four times (n = 4) under similar conditions with comparable results.

ACE2 is a transmembrane zinc-containing metalloenzyme of 805 amino acid residues, with seven N-glycosylation sites and three disulfide bridges (Swiss-Prot code Q9BYF1). It is a single-pass type I integral membrane glycoprotein, orientated with the N-terminus and the catalytic site facing the extracellular space as an ectoenzyme. ADAM17, a member of the “A Disintegrin And Metalloproteases” (ADAMs) family, is known to cleave a variety of membrane-anchored proteins including ACE2. The cleaved form of ACE2 is called circulating ACE2 (cACE2), and is released as an enzymatically active ectodomain of ACE2 into the plasma that requires zinc cation for its activity [25]. The amino acid residues hydrolyzed by the peptidase reveals a potential consensus sequence, **P-X_1-3 residue_-P-**↓**-X _(hydrophobic/basic)_**. The sequence of the VE-cadherin is depicted in Figure 2a and its adhesive extracellular domain is highlighted in yellow. We analyzed the potential sites of cleavage by ACE2 and found two consensus sites of cleavage at the peptide bonds (**^266^P-F^267^**) and (**^321^PMKP–L^325^**) that are highlighted in red in Figure 2a. Therefore, we hypothesized that VE-cadherin could represent a target for ACE2.

To investigate this possibility, we used a commercially available human recombinant ACE2 enzyme (hrACE2) and the hrED of VE-cadherin purified as described above. As shown in Figure 2b, the analysis of the hrACE2 ectoenzyme by SDS-PAGE and immunoblotting using the antibody directed against the ectodomain of the enzyme showed that one band was detected with the predicted molecular mass of 110 kDa. The purified protein rACE2 was then applied to the hrED of VE-cadherin in vitro, in the recommended conditions using the prediluted rACE2 in 50 mM 2-(N-Morpholino)-ethane sulfonic acid (MES), pH 6.5, 300 mM NaCl, 10 µM ZnCl_2_, 0.01% Brij 35. Increasing concentrations of hrACE2 were added to the extracellular domain of VE-cadherin overnight at room temperature. The mixture was then analyzed by SDS-PAGE and westernblotted with the mouse monoclonal antibody (BV9) and the rabbit polyclonal EC1 antibody directed against the extracellular domain of VE-cadherin (Figure 2c,d). The results show that after rACE2 incubation the intensity of the immunoreactive band detected either with the clone BV9 or with the anti-EC1 antibody decreased in a dose-dependent manner from 0.1 to 4 ng/mL. We quantified the densitometric absorption of the immunoreactive band using Image J software 1.54f (Figure 2e,f). The minimum threshold concentration of hrACE2 ectoenzyme required for the partial cleavage of VE-cadherin was 0.5 ng/mL, which corresponds to the concentration found in blood.

As expected, we found that VE-cadherin was a substrate for hrACE2 while the probability for hrACE2 to cleave VE-cadherin was unlikely because it is a carboxypeptidase. But we can speculate that during the cytokine storm induced by SARS-CoV-2 infection, VE-cadherin undergoes conformational rearrangements such as those described for phosphoproteins. Indeed, we have previously shown that VE-cadherin became a phosphoprotein in response to several cytokines, a process that is associated with disruption of cell-cell junctions [13]. Because phosphate groups are highly negatively charged, phosphorylation of VE-cadherin altered charge and thus conformational changes in the protein that favor its proteolytic cleavage [26]. Overall, the data from the present study suggest that the extracellular adhesive domain of VE-cadherin can be an unusual target for the ecto-metallopeptidase ACE2.

### 2.3. Circulating ACE2 Was Detected in Blood Samples from COVID-19 Patients

In order to know if such a process could occur in vivo, we first assessed the presence of cACE2 in COVID-19 patients with a mild or severe COVID-19 disease, since it was reported that the concentrations of cACE2 correlate with the systemic inflammation [27]. All nine patients studied here had COVID-19 infection that was confirmed by nasal reverse transcription coupled to gene-specific polymerase chain reaction. Blood samples were taken under fasting conditions at the diagnosis of infection, before systemic treatment was proposed. Severe forms of COVID-19 (n = 5) were defined as respiratory failure requiring mechanical ventilatory assistance, while mild forms (n = 4) were defined as patients requiring hospitalization but without mechanical ventilatory assistance. Patient characteristics are described in Table 1. The first group comprises 4 patients without admission to the intensive care unit (ICU) while in the other group, there were 5 patients admitted to ICU with respiratory failure at admission, O2-supplementation and orotracheal intubation.

**Figure 3:**
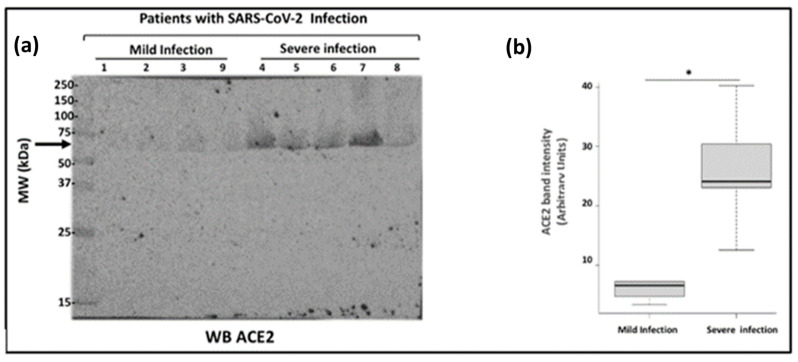
Analysis of circulating ACE2 in blood from patients with SARS-CoV-2 infection. (**a**) Blood samples from COVID-19 patients were analyzed by SDS-PAGE and immunoblotting. Blood samples were diluted serially from 1 to 10 followed by a dilution of 1 to 2.5. 15 μL of sample preparations were loaded onto an SDS-PAGE and then transferred onto a nitrocellulose membrane. Proteins on the blots were visualized by Ponceau staining. The positions of molecular mass standards (in kDa) are shown at the left. The membranes were incubated with the anti-ACE2 antibody (5 μg/mL) followed by incubation with the anti-mouse peroxidase antibody. The arrow on the left-hand side shows the immunoreactive band corresponding to cACE2. (**b**) Densitometry analysis of the immunoreactive band corresponding to cACE2 to compare the groups (n = 4 for mild infection and n = 5 for severe infection). Error bars represent mean ± SE of means. p values from analysis of variance were assessed using the Mann-Whitney test (* *p* < 0.05). These experiments were repeated at least four times in a similar configuration.

The blood serum samples were diluted serially in a detergent-containing buffer according to our published protocol [13]. We chose to analyze the samples by western blot because we wanted to determine the apparent molecular mass of the expected cACE2. The diluted sera were analyzed on a 10% SDS-PAGE and immunoblotting with the anti-human ACE2 ectodomain monoclonal antibody. Figure 3a depicted the immunoblot for the ectodomain of cACE2 using the serum of patients with SARS-CoV-2 infection. The presence of only one species of cACE2 with a molecular mass of 75 kDa was highly detectable in patients with severe infection while it was weak in patients with mild disease. Densitometry analysis of the immunoreactive band corresponding to cACE2 showed a significant difference between the group of severe infection (n = 5) (24.08 ± 3.62) compared to the group with mild infection (6.67 ± 0.59) (n = 4) (*p* = 0.014) (Figure 3b). These data are consistent with the literature that showed a soluble form of ACE2 was detected in blood from COVID-19 patients with a severe infection [27].

### 2.4. Analysis of Soluble VE-Cadherin in Blood Samples from COVID-19 Patients

**Figure 4:**
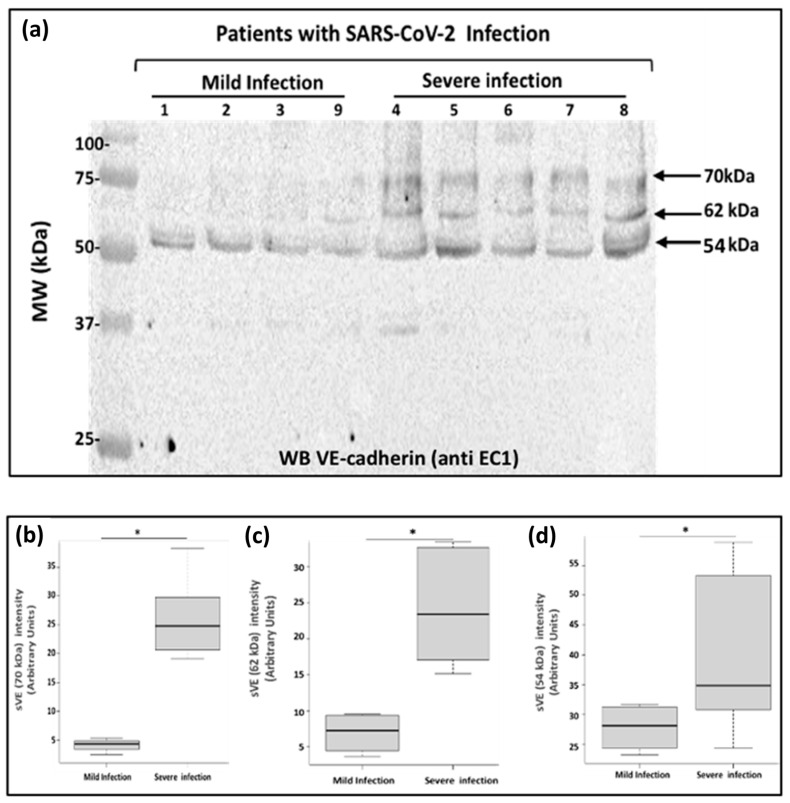
Analysis of the soluble forms of VE-cadherin in COVID-19 patient blood. (**a**) Blood samples from COVID-19 patients were analyzed by SDS-PAGE and immunoblotting. Proteins on the blots were visualized by Ponceau staining. Blood samples were diluted serially from 1 to 10 followed by a dilution of 1 to 5 (total dilution 1:50). 15 μL of sample preparations were loaded onto the gel and then immunoblotted with the anti-VE-cadherin antibody (EC1 1:250) overnight at 4 °C followed by an incubation with the goat anti-rabbit peroxidase antibody (1:2000). Arrows indicate the apparent molecular masses of the immunoreactive VE-cadherin. The positions of molecular mass standards (in kDa) are shown at the left. This experiment is representative of three independent experiments. (**b**–**d**). Densitometry analysis to compare the groups (n = 4 for mild infection and n = 5 for severe infection) for the three different forms of soluble VE-cadherin 70 kDa (**b**), 62 kDa (**c**), 54 kDa (**d**). The differences between patient groups were assessed as being significant using Mann-Whitney analysis (* *p* < 0.05). These experiments were repeated at least four times in a similar configuration.

Next, we investigated if soluble VE-cadherin could be detected in the patient’s blood and if yes, we wanted to determine its molecular weight. We thus analyzed the sera from the same patients (Table 1) by westernblotting using the antibody directed against VE-cadherin extracellular domain EC1. As a result, we demonstrated that several forms of soluble VE-cadherin were detected in patients with severe disease while only one form was found in patient’s blood with mild disease (Figure 4a). In the blood from patients having a severe infection, the apparent molecular masses of VE-cadherin were 70 kDa (4.14 ± 0.56 vs. 26.51 ± 3.72; *p* = 0.015), 62 kDa (6.89 ± 1.45 vs. 24.33 ± 3.53; *p* = 0.015), and 54 kDa (26.44 ± 3.83 vs. 36.64 ± 7.76; *p* = 0.015) (Figure 4b). The immunoreactive band with an apparent molecular mass of 54 kDa was the only one expressed in the patients with mild infection. Our data provide evidence for multiple forms of the truncated VE-cadherin depending on the severity of the disease. In rheumatoid arthritis and HAE, we previously showed a 90 kDa band corresponding to the full length of the extracellular domain of the protein which was a marker related to disease activity score [9,14]. The results presented here need further investigations to better understand whether these different soluble forms of VE-cadherin might represent good clinical biomarkers to classify the patients.

### 2.5. Proposed Model Scheme for Endothelial Dysfunction following SARS-CoV-2 Infection

**Figure 5:**
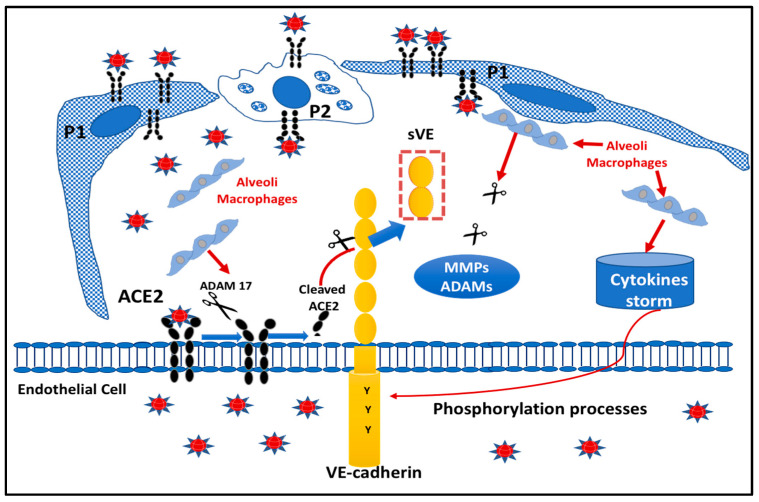
Proposed model of Pulmonary Endothelial dysfunction in COVID patients: In the pulmonary environment, the ECs are surrounded by pneumocytes (P1, and P2) and macrophages that are responsible for the cytokine storm. ACE2 is present on ECs as a transmembrane protein whose catalytic site is outside the cells (ectopeptidase). ACE2 can be cleaved by ADAM 17 and leads to the generation of a circulating active form of ACE2. VE-cadherin is a transmembrane protein exclusively expressed in ECs, which can be subjected to post-translational modifications including tyrosine phosphorylation upon cytokine challenge. This covalent modification will lead to a conformational change in the protein structure, which presents a high sensitivity to proteases. Thus, the ACE2 enzyme will act directly to its potential site of cleavage generating the fragments of VE-cadherin seen in the patient’s blood.

We have illustrated in Figure 5 a proposed scheme of the COVID-19 microenvironment at the pulmonary level. The very close interaction between pneumocytes (P1, P2), alveolar macrophages, and EC of pulmonary capillaries led us to propose that the infection by the virus creates an environment where several molecules (cytokines, proteases) can induce structural changes in the cytoplasmic domain of VE-cadherin (e.g., TNF) such as by phosphorylation processes. Tyrosine phosphorylation will change the charge of the protein, resulting in alterations of the affinity of VE-cadherin homophilic interactions of the extracellular domain [7]. This post-translational modification renders the protein more susceptible to proteases such as for cell-surface associated receptor tyrosine kinases [26]. Thus, it is tempting to suggest that the protease ACE2 could be released from the membrane of ECs by ADAM17, to further cleave the extracellular adhesive domain of VE-cadherin at the two sites for active cACE2.

## 3. Discussion

Recent clinical reports [4,5] coupled with previous publications [5,6] provide strong evidence to consider that the EJ structure is a central target that leads to damage to ECs, endothelialitis, coagulopathy and microcirculation dysfunction in SARS-CoV-2 infection. The findings presented here demonstrate for the first time that VE-cadherin, a ubiquitous endothelial cell marker expressed at the adherens junctions of ECs, is modulated in vitro by the angiotensin-converting enzyme-2 (ACE2). The most compelling results arise from our studies showing the two sites of cleavage at the positions ^256^P-F^257^ and ^321^PMKP-^325^L in VE-cadherin adhesive ectodomain. The accessibility of these sites of cleavage by the ectoenzyme ACE2 requires conformational changes in the structure of VE-cadherin, which are a consequence of phosphorylation by cytokines (i.e., TNF alpha; Bradykinin, VEGF, etc., …) released following SARS-CoV-2 infection [6]. Of interest, previous studies have shown that ACE2 can also hydrolyze several peptides such as dynorphin A (1–13), apelin-13, des-Arg9 bradykinin, β-casomorphin and ghrelin [28]. The capacity of ACE2 to hydrolyze substrates other than Ang II suggests that ACE2 may have other functions that remain to be explored [28]. Varga et al. showed that SARS-CoV-2 infection causes vascular inflammation in different organs [4]. In contrast to normal conditions, the endothelium and more precisely VE-cadherin become activated by circulating inflammatory mediators, which could account for the vascular symptoms observed in patients having a mild or severe forms of COVID-19 disease. Given the central role of ACE2 in SARS-CoV-2 infection, and the central role of VE-cadherin in the integrity of the endothelium, we hypothesize that the vascular symptoms in the “Long COVID” might be due to VE-cadherin attack. Our hypothesis is supported by our measurements of several soluble forms of VE-cadherin in the blood of patients with COVID-19. Indeed, we detected for the first time three different fragments of truncated VE-cadherin with apparent molecular mass of 70, 62, and 54 kDa in the blood of patients presenting severe COVID-19 disease. In comparison, only the 54 kDa was found in patients with mild COVID-19 disease. The putative sites for ACE2 cleavage located in EC2 and EC3 ectodomain of VE-cadherin could produce the 62 and 54 kDa forms that we identified [29]. However, we cannot exclude the involvement of other proteases such as neutrophil elastase, which was recently published in COVID-19 patients [30], as well as serine proteases such as proteinase 3, cathepsins, and ADAMs from neutrophils [31]. Our description of VE-cadherin cleavage in COVID patients is original and novel and the future quantification of soluble VE-cadherin fragments will provide a blood test for personalized COVID-19 patient care, management and follow-up. The soluble VE-cadherin has already been recognized as a marker of different vascular pathologies including atherosclerosis, rheumatoid arthritis and more recently in Obstructive Sleep Apnea Syndrome [32,33,34]. Further investigations will be of major importance to determine the association between several forms of soluble VE-cadherin, vascular endothelial dysfunctions and coagulopathies in Long COVID-19 [1].

Taking into account the local pulmonary environment and the damage to other organs in COVID-19, we hypothesize that the combined actions of ACE2 and/or other proteases might be at the origin of vascular lesions. ACE2 has been described as a transmembrane enzyme [35], whose role was perfectly described as being the receptor for SARS-CoV-2 but its functions as a soluble effector remain to be defined [36]. Previous studies have shown soluble form of ACE2 in human, murine fluids and airways and its shed ectodomain comprises its carboxypeptidase activity [37]. This cACE2 form is normally found in the blood of healthy donors at low levels. Here we demonstrate that cACE2 was detected in blood from COVID-19 patients at a higher level in patients with severe infection. These results are in agreement with previous data reporting that cACE2 was correlated with the severity of COVID-19 disease [23,38]. In recent reports from the literature, cACE2 is more considered as a prognostic marker in monitoring diseases as it can be a potential therapeutic target to inhibit SARS-CoV-2 entry into cells [39] as well as the therapeutic use of human recombinant ACE2 [40,41]. In those publications, the quantification of cACE2 in plasma or serum is based on enzyme activity (relative fluorescence units), with the conversion to a concentration of enzyme by reference to a standard curve [42]. In comparison, here we demonstrate directly the presence of cACE2 in blood from COVID-19 patients using western blot. Indeed, this technique uses denaturating conditions. Furthermore, the blood samples were treated according to our patent that provided a method to increase the sensitivity of detection of serum protein biomarkers by diluting the serum in a detergent-containing buffer to remove the masking influence of the lipoproteins and other lipids [43]. The antibody used for the detection of cACE2 in the blood was the commercially available antibody used for recognition of hrACE2 (see Figure 2). All the samples were analyzed on the same gel so the exposure was the same and it was possible to compare the two groups of patients with the immunodetection of only one band associated with severe infections. To the best of our knowledge, this is the first description of cACE2 detection in blood in which the apparent molecular mass corresponds to the 740 amino acid sequence of the ectodomain of ACE2 [44]. Nonetheless, the amount of cACE2 has not been quantified in our study because the specificity of the antibodies might differ based on the glycosylation processes. In future studies, in addition to western blots, it would be of interest to develop immunoquantification of cACE2 based on different antibodies that could recognize all the forms of the enzyme in patient’s blood.

To conclude, future fundamental research is still needed to explore whether SARS-CoV-2 targets VE-cadherin through proteases or other activated pathways within the SARS-CoV-2 environment. However, the results provided in this study regarding the detection of both cACE2 and soluble VE-cadherin in blood are very encouraging for predicting gradations in the severity of SARS-CoV-2 infection. Continuing efforts to elucidate whether the size of sVE fragments in the blood of COVID-19 patients would be of prime significance to provide clinicians with quantifiable information on which to base decision-making to precisely target the actors of “infection” and “damage” caused by COVID-19 to allow “personalized” medicine. Altogether, these data shed light on the possible vascular “Long COVID-19” disease.

## 4. Materials and Methods

### 4.1. Antibodies

Recombinant Human ACE2 Protein (933-ZN) was purchased from R&D Systems (Minneapolis, MN, USA) and stored in a buffer according to the manufacturer’s instructions. Monoclonal mouse anti-human ACE2 Antibody (MAB933) was purchased from R&D Systems (Minneapolis, MN, USA). Human recombinant VE-cadherin extracellular domain (EC1-5) was produced in the laboratory. Peroxidase AffiniPure Goat Anti-Mouse IgG (H + L) (AB-2338447), Peroxidase AffiniPure Goat Anti-Rabbit IgG (H + L) (AB-2307391), and Cy3™ AffiniPure Goat Anti-Mouse IgG (H + L) (AB-2338680) were all purchased from Jackson Immunoresearch (Ely, Cambridgeshire, UK). Hoechst solution (33,258) was purchased from Sigma Aldrich. The mouse monoclonal anti-human VE-cadherin antibody (clone BV9) and the rabbit polyclonal anti-EC1 anti-human VE-cadherin antibody were produced in the laboratory.

### 4.2. Cell Culture

Human embryonic kidney cells (HEK293-EBTNA were grown as described in [13].

### 4.3. Protein Extracts Analysis

Protein extraction, SDS-PAGE and Immunoblotting were performed according to [13]. Briefly, the human glycosylated VE-cadherin extracellular domain was produced from human embryonic kidney cells (HEK293-EBTNA) according to a previously published protocol [24]. Proteins were subjected to electrophoresis on SDS-PAGE 10%, and then transferred onto a 0.45 μm nitrocellulose membrane. Membranes were blocked with milk, incubated with primary antibodies and corresponding HRP-conjugated secondary antibodies. Immunoreactive proteins were visualized by Bio-Rad ChemiDoc™ with ECL reagent (Bio-Rad Laboratories, 92430 Marnes-la-Coquette, FRANCE) and were quantified using densitometry with Image-J software (NIH, Bethesda, MD, USA). All Western blots were representative of at least three to four independent experiments with similar findings.

### 4.4. Patient Sera

Samples were taken from a serum bank that was available at Grenoble University Hospital. Analyses were performed on samples from 9 patients with COVID-19. Five of them had a severe form of COVID-19 with a passage in resuscitation and intubation. The other 4 patients had mild forms, requiring only oxygen therapy (see Table 1).

### 4.5. Statistical Analysis

All the experiments were repeated at least three times. Data are expressed in arbitrary units as the mean ± SD of at least 3 identical experiments and were compared using Mann-Whitney test. For all tests, *p* values less than or equal to 0.05 were considered significant.

## Figures and Tables

**Table 1:** Characteristics of COVID-19 patients with either a mild form (1, 2, 3, 9) that required only oxygen therapy or a severe form (4, 5, 6, 7, 8) that required resuscitation and intubation and admission to ICU (ICUA). The age, background and existing treatments of the patients are also summarized. For patients 1, 2, 3, 9 no mechanical ventilatory assistance was required. For patient 5, the prior treatment was not known.

Patients	Age	Management Protocol	ICUA	Background	Treatments
1	88	-	No	Hypertension, hyperuricaemia, Benine Prostate Hypertropnhia, chronic renal failure	Allopurinol, bétaxolol, fénofibrate, lercanidipine, alfuzosine
2	80	-	No	Hypertension, hyperuricaemia, Benine Prostate Hypertropnhia, chronic renal failure	bisoprolol, amiloride
3	67	-	No	OSAS, Benine Prostate Hypertropnhia, Rotator cuff surgery, sigmoidectomy for diverticular disease	none
9	63	-	No	Appendicitis	none
4	65	Mechanical ventilatory assistance, Prone position, NO/Pulmonary embolism thrombolysis	Yes	Deep vein thrombosis, obesity, OSAS, Umbilical hernia with prosthesis	none
5	86	Atrial Fibrillation, Pneumopathy (S aureus and enterobacter)	Yes	Hypertension, Atrial fibrillation treated with anti-clotting medication	-
6	52	Mechanical ventilatory assistance, Prone position, Pumonary embolism	Yes	Hiatal hernia, clavicle fracture	none
7	53	Mechanical ventilatory assistance, Prone position, Cardiopulmonary bypass	Yes	Hypertension, dyslipidemia, obesity	none
8	73	Mechanical ventilatory assistance, Prone position	Yes	Hypertension, diabetes	bisoprolol, vildagliptine/metformine, gliclazide, kardegic, pravastatine

## Data Availability

Not applicable.
